# Revealing the pharmacological effect and mechanism of darutoside on gouty arthritis by liquid chromatography/mass spectrometry and metabolomics

**DOI:** 10.3389/fmolb.2022.942303

**Published:** 2022-08-24

**Authors:** Jing Wang, Yan-Chun Sun

**Affiliations:** ^1^ School Hospital, Harbin University of Science and Technology, Harbin, China; ^2^ Heilongjiang River Fisheries Research Institute of Chinese Academy of Fishery Sciences /Laboratory of Quality & Safety Risk Assessment for Aquatic Products (Harbin), Ministry of Agriculture and Rural Areas, Harbin, China

**Keywords:** metabolomics, biomarker, pathways, target, liquid chromatography, mass spectrometry

## Abstract

Darutoside is a diterpenoids compound with significant anti-inflammatory activity, however the pharmacological action and mechanism are still unclear. Metabolomics strategy was used to uncovering the pharmacological action and effective mechanism of darutoside against acute gouty arthritis rats. Liquid chromatography coupled with mass spectrometry technique was performed to explore the serum metabolites and potential pathways. We found that darutoside can up-regulate the level of glutamate, alanine, chenodeoxycholic acid, 1-methyladenosine, aspartic acid, citric acid, and down-regulate the level of valine, isoleucine, glutamine, alanyl-threonine, pyruvic acid, gamma-aminobutyric acid, uric acid. Metabolic pathway analysis showed that the therapeutic effect of darutoside was involved in amino acid metabolism, sugar metabolism, fatty acid metabolism, energy metabolism, purine metabolism and butanoate metabolism. It indicated that darutoside protect against acute gouty arthritis by regulating the expression of the key protein targets. It revealed that the mechanism of darutoside on acute gouty arthritis, which may be leading to the changes of serum metabolites, metabolic pathways and key protein targets to improve immune system response, inhibit oxidative stress and inflammatory response. It provides a novel method for molecular mechanisms of natural product in the disease treatment.

## Introduction

Acute attacks of gout as a kind of inflammatory disease in arthritis field bringing a lot of pain to patients is mainly caused by the deposition of uric acid and generation of monosodium urate (MSU) crystals within joints and periarticular soft tissues (Kim et al., 2003; Jordan et al., 2007; Mandell, 2008). In the early stages of the disease, acute attacks usually focuses on monoarticular, foot or great toe. In company with the development of disease, urate (UA) deposition is increased in body leading to recurrent attacks may occur in any joint. If the patient has not been effectively treated for a long time, the serum urate level will increase drastically (Dalbeth et al., 2019; Singh, 2019). Hyperuricemia is the major risk factor, which may originate from purine metabolism turbulence and UA-induced impairment of renal excretion, and was also related with some chronic disorders such as heart failure, chronic kidney disease, coronary artery disease, and metabolic syndrome (Kuo et al., 2015; Li et al., 2018). The prevalence of gout is constantly increasing worldwide, more than eight million adults suffer from gout in the US alone. In light of the European League Against Rheumatism (EULAR) report, approximately 1–2% adult Western population present symptomatic gout, and rising to 7% in those aged over 65 years (Saigal and Agrawal, 2015; Singh and Gaffo, 2020). Modern studies were found that IL-1β as a crucial inflammatory mediator induced may be a potential therapeutic target for the treatment of acute gouty arthritis (Cronstein and Sunkureddi, 2013; Fang and Waizy, 2013). Currently, reducing urate therapy and inflammatory response are the goal of the clinical therapy of gout in clinical practice. Anti-inflammatory agents, colchicine, NSAIDs, corticosteroids, and anti-IL-1β biologics, are widely applied for the treatment of gout flare. However, they are not authorized by United States (US) Food and Drug Administration (FDA). Because of the discrepancy between treatment recommendations and clinical practice, many gout patients require to be more monitored for avoiding suboptimal management and unpredictable side effects such as skin rash, fever, gastrointestinal puncture, liver and kidney function damage (Khanna et al., 2014; Fields, 2019). With the continuously growing incidence, searching accurate diagnosis technology in early stage and best treatment is urgently needed.

Since the outbreak of COVID-19 in 2019, natural products have become an important resource for seeking potential therapeutic candidates against disease by the global research community’s attention (Yang Y. et al., 2020). Darutoside is one of diterpenoid ingredient, however, the pharmacological action and effective mechanism protecting against rats with acute gouty arthritis has hardly been reported at present (Son et al., 2005; Su et al., 2014; Guo et al., 2018a; Guo et al., 2018b; Sang et al., 2018). Metabolomics as a member of systems biology aims to depict the dynamic metabolic profile of endogenous metabolites with low molecular weight throughout the biological system using modern analytical technologies such as nuclear magnetic resonance (NMR), gas chromatography-mass spectrometry (GC-MS) and liquid chromatography-mass spectrometry (LC-MS) coupled with multivariate analysis. It could bring out a forceful platform for discovering differential biomarkers and vital biochemical pathways to monitor and diagnose disease progression, improve treatment and prediction in complex systems through evaluating the level variations of metabolic biomarkers (Xie et al., 2017; Sun et al., 2018; Xie et al., 2019; Zhang et al., 2019a; Zhang et al., 2019b; Du et al., 2020; Ren et al., 2020; Yang J. et al., 2020; Zhang et al., 2020; Zeng et al., 2021). Network Pharmacology that is one of the organic combinations of systems biology and bioinformatics, integrates multi-disciplinary technical content including biology, multi-directional pharmacology, and computational biology, and the analysis of real data of genes, proteins, diseases, drugs and others obtained in the database and laboratory to establish a multi-level network from a systematic and overall perspective for revealing the mechanism of multi-molecular drugs acting on the body. Now, it has become a powerful tool for exploring complex diseases and disclose the complex relationship among proteins, diseases and drugs, which is completely important for explaining the underlying mechanism of TCM.

Rats were injected by sodium urate emulsion into joint cavity leading to neutrophil infiltration, synovial inflammatory cell infiltration, uric acid deposition, local tissue necrosis, synovial tissue hyperplasia, and inflammatory factor release, which the pathological manifestations are very similar to clinical. The combination of metabolomics and network pharmacology can link endogenous metabolites with disease targets, thereby further revealing the molecular mechanism of TCM with multi-component and multi-target characteristics (Zhang et al., 2018; Fan et al., 2020). Recently, some researchers have successfully used the integrated metabolomics and network pharmacology strategies to quest the interaction between organisms and drugs, which has brought great inspiration to the mechanism research of darutoside. The purpose of current study is to explore the pharmacological action and effective mechanism of darutoside against sodium urate-induced acute gouty arthritis rats by integrated LC-MS based serum metabolomics strategy coupled with network pharmacology. Further, the compound-target-metabolite network was constructed to seek the key targets of acute gouty arthritis by natural compounds darutoside.

## Methods

### Reagents

MSU were purchased from the Jiancheng Institute of Biotechnology (Nanjing, China). Pentobarbital sodium, paraformaldehyde and physiologic saline solution were purchased from Shanghai Chemical Reagent Co. (Shanghai, China). The darutoside with more than 97.4% purity (batch number: BCBS7438) were obtained from Chengdu Cloma Biological Co., Ltd. (China) and the HPLC chromatography was shown in Supplementary Figure S1. Enzyme-linked immunosorbent assay (ELISA) kits for interleukin-8 (IL-8), interleukin-1β (IL-1β) and tumor necrosis factor-α (TNF-α) assay were purchased from Assay R&D Systems. Nuclear factor kappa-B (NF-κB) and interleukin-10 (IL-10) were purchased from Biowest (Nuaillé, France) and HyClone (Logan, Utah, United States), respectively. Blood uric acid (UA) was bought from Chondex (Redmond, WA, United States). LC-MS grade acetonitrile (ACN) and methanol (MeOH) were purchased from Merck (Darmstadt, Germany), and formic acid (FA) was purchased from Sigma Chemicals Ltd. (St. Louis, MO, United States). Double-distilled water was purified using a Millipore water purification system (Millipore, Bedford, MA). Leucine enkephalin as a standard substance with the purity more than 99.2% were obtained from Hyclone (Logan, UT, United States). Other reagents used were of analytical grade and were purchased locally.

### Animal experiments and drug administration

Forty-eight male Wistar rats (8 weeks old, weighing 150 ± 10 g) were obtained from Experimental Animal Center of Beijing Academy of Medical sciences. Animals were utilized and processed during procedures of experiment in light of the National Institutes of Health guidelines and ethical regulations for the care and use of laboratory animals of School Hospital, Harbin University of Science and Technology, which aims to make minimize animal numbers and suffering. Rats were acclimatized 7 days to adjust to external environment before any experimental operation, which were housed in plastic cages with a temperature of 24 ± 2°C, humidity of 55 ± 3%, 12/12 h light/dark cycle (8:00 a.m. to 8:00 p.m.), and free access to diet and tap water. Health state of the rats was monitored and recorded every day during the housing period. Rats were randomly divided into control group (CON group), model group (MOD group), model rats with colchicine treatment group (MOD + COL group), and model rats with darutoside treatment group (MOD + DAR group) with 15 rats in each group.

The first dose was administered to each group 2 h before modeling. Rats in MOD + DAR group and MOD + COL group were respectively given 40 mg/ml darutoside and 0.15 mg/ml colchicine at a dose of 10 ml/kg one time by oral administration. Rats in CON and MOD group given equal doses of distilled water. MSU are crushed, then was added to a beaker with physiological saline and Tween 80 at a ratio of 1:9:1, heated and stirred to prepare a sodium urate emulsion. Two hours later, the right ankle joints of the rats in every group were moderately bent and locally disinfected with alcohol. The syringe was tilted 45° to inject about 150 μL of 100 g/L sodium urate emulsion into the ankle cavity of rat in MOD group, MOD + COL group, MOD + DAR group, and then stretched and rotated for 1min. Rats in CON group were injected with 150 μL 10% Tween 80 prepared by normal saline into the joint cavity (Lyu et al., 2019; Goo et al., 2021; Li et al., 2022). Contralateral articular capsule protrusion was used as the model preparation standard. At 6 h and 22 h after modeling, each group was given corresponding drugs again. The changes of various indexes were observed 24 h after modeling.

### Sample collection and preparation

After 24 h of the sodium urate emulsion injection, all the animals were anesthetized by 4% pentobarbital sodium (0.3 ml/100 g body weight) in intraperitoneal injection manner. Blood samples were collected and centrifuged at 4,500 rpm for 10 min to separate the serum without hemolysis. Serum sample were stored at − 80°C for the biochemical parameters and metabolomics analysis. Then, rats were sacrificed and synovial tissues were collected in 4% paraformaldehyde solution for histological analyses. For serum metabolomics analysis, serum sample were firstly thawed at room temperature, and then were extracted with methanol in ratio of 1:3 (v/v) in order to precipitate protein. The mixture was vortexed for 1 min and centrifuged at 3,500 rpm for 10 min. The obtained supernatants were evaporated to dryness at 37°C, and the residues were added 500 μL initial mobile phase to re-dissolve. After centrifuging at 10,000 rpm for 5 min under 4°C, the prepared samples were filtered through 0.22 μm micropore filter for detection (Ugarte et al., 2012; Wen et al., 2020; Su et al., 2021). Furthermore, all samples which was taken 10 μL from each were mixed together as quality control (QC) samples, then processed in the same method as above. Due to containing the most data in all groups, QC sample has potential to validate the stability of LC-MS system.

### Conventional pharmacodynamic evaluation

After modeling, the changes of food intake, coat color, physical activity level and mental state were observed and recorded. The same position of the right ankle joint of all rats was marked, and then the volume changes of the marked area before modeling and 24 h after the modeling were measured by a toe measuring instrument. Meanwhile, the thickness changes of the right ankle joint injected with sodium urate before modeling and 24 h after the modeling were measured by vernier caliper. The difference of volume and thickness alteration were calculated. Blood concentrations of IL-8, TNF-α, IL-1β, NF-κB, IL-10 and UA were tested according to commercially specific ELISA kits following the manufacturer’s instructions. Intravenous blood sample were collected into the anticoagulant tube (sodium citrate 1:4) and immediately inverted and mixed.

### Instrument condition of liquid chromatography-mass spectrometry

Serum metabolic analysis was achieved on an Agilent 6,550 iFunnel Q-TOF LC/MS system (Agilent Technologies, Santa Clara, CA, United States). Each serum samples were separated though an ACQUITY UPLC HSS T3 column (100 mm × 2.1 mm, 1.7 μm, Waters, Milford, MA, United States) at the flow rate of 0.30 ml/min under 30°C. The injected volume is 5 μL. Mobile phase A and Mobile phase B are consist of water containing 0.1% formic acid and ACN containing 0.1% formic acid, respectively, which run according to a linear elution gradient as follows: 5%B over 0–80%B over 6–8 min, 80% up to 90% B over 8–10 min, 90% down to 5%B 10–12 min, 5%B over 12–14 min. Both positive (ESI+) and negative (ESI-) mode electrospray ionization sources were applied in MS and the optimized parameters were set as follows: electrospray capillary voltage is 4.0 kV in ESI + mode and 3.0 kV in ESI- mode, ion spray voltage is 4.5 kV, ion source temperature is 450°C, curtain gas is 30 psi, declustering potential (DP) is 65 V, collision energy (CE) is 40 eV, and collision energy spread (CES) is 15 eV, gas temperature is 240°C, gas flow rate is 15 L/min, and nozzle voltage is 2100 V in both ion mode. Mass range is captured from m/z 50 to 1,500 in the centroid mode. In order to assure the accuracy of instrument, 0.15 ng/ml leucine enkephalin as LockSpray interference that *m/z* is 556.2771 in ESI + ion mode and *m/z* is 554.2614 in ESI- ion mode was analyzed. Before analysis, QC samples were detected for six times to balance the LC/MS system, and subsequently every eight samples were utilized to observe whether the systematic error of the whole experiment is within the controllable range.

### Data extraction and pattern recognition analysis

Original data were imported into MakerLynx within MarkerLynx XS Version 4.1 software (Waters Co., Milford, MA, United States) for peak data extraction, noise reduction, matching, alignment and detection. After unit variance scaling and the mean-centered method management, the information of each analyte such as identity (ID), m/z, peak area, retention time (Rt) and ion strength was gained, which the main parameters were as follows: m/z 50 to 1,500, Rt range 1–10 min, mass tolerance 0.1 Da, and noise elimination level 5. The data matrices were imported to SIMCA-14.1 software (Umetrics, Umea, Sweden) for multivariate statistical analysis such as principal component analysis (PCA) and orthogonal projection to latent structures-discriminant analysis (OPLS-DA) analysis. PCA as an unsupervised method of pattern recognition approach is used to generally describe the maximum variation between different groups of metabolites. OPLS-DA as a supervised method of pattern recognition approach only be utilized for screening differentially expressed metabolites between the two groups. In OPLS-DA mode, the variance of the response variable (R2Y) is close to 1 and the variance for modeling in cross-validations (Q2) is more than 0.5 at the same time suggesting that the modes possess good explanatory and predictive ability. The permutation tests (*n* = 100) were employed to prove the predictive ability of modes, which indicates the goodness of fit and better predictability of the OPLS-DA mode when all the R2 and Q2 values were lower than that in the permutation tests. The S-plot from the OPLS-DA mode explore the intrinsic variables that provide significant contribution to the metabolic separation, which the variable importance in the projection (VIP) value more than 1.0 can be deemed as potential biomarkers.

### Biomarkers identification and pathway analysis

Variables with VIP more than 1.5 were eventually submit to Student’s t-test, and it was regarded as statistically significant when *p*-value < 0.05. The screened out candidate variables were tentatively identified according to the online METLIN database (http://www.metlin.scipps.edu), HMDB database (http://www.hmdb.ca/), SMPDB (http://www.smpdb.ca/) and KEGG (http://www.kegg.com/), and further affirmed as endogenous metabolites by MS/MS fragment, literature and standard reference substance comparison. Heatmaps was used to visualize the serum level changes though different colors in the regulatory manners among different group. Metabolic pathway analysis of the identified potential biomarkers was performed with MetaboAnalyst 4.0 (http://www.metaboanalyst.ca) and Kyoto Encyclopedia of Genes and Genomes (KEGG; http://www.kegg.jp/) based on the pathway library of *Rattus norvegicus* (rat).

### Network pharmacology analysis

For fully understandings of the valid mechanism regarding darutoside treating acute gouty arthritis, the network of “compound-target-metabolite” was built. The targets of darutoside were collected from TCMSP (http://lsp.nwu.edu.cn/tcmsp.php) and Pharm Mapper (http://lilab.ecust.edu.cn/pharmmapper), which were screened by the condition that oral bioavailability (OB) ≥ 30% and a drug-likeness (DL) > 0.18. Full name of protein targets were transformed into protein IDs by UniProt database (https://www.uniprot.org/). Gene Cards (https://www.genecards.org/) and MBROLE 2.0 (http://csbg.cnb.csic.es/mbrole2) database were used to explore the protein targets for potential metabolites concerning darutoside against rats with acute gouty arthritis. The biological interaction network of compound-target-metabolite was visualized using Cytoscape 3.7.1 software (Cytoscape Consortium, California, United States). Protein-protein interaction network database (DIP, http://dip.doe-mbi.ucla.edu/dip/Main.cgi and IntAct, https://www.ebi.ac.uk/intact/) was employed to select vital protein targets about darutoside efficacy.

### Statistical analysis

All the data were disposed using SPSS software (version 16.0; SPSS, Chicago, IL, United States) and described as the mean ± standard error of the mean (SEM) way. Differences between two or more groups were analyzed by ANOVA or unpaired Mann-Whitney U test or Student t test, which the samples were done two or three independent assays. *p* value less 0.05 was believed statistically significant. Graphics in experiment process were produced though Prism software (version 5.0; GraphPad Software, La Jolla, CA, United States).

## Results

### Pharmacological effect of darutoside on conventional indicators

**FIGURE 1:**
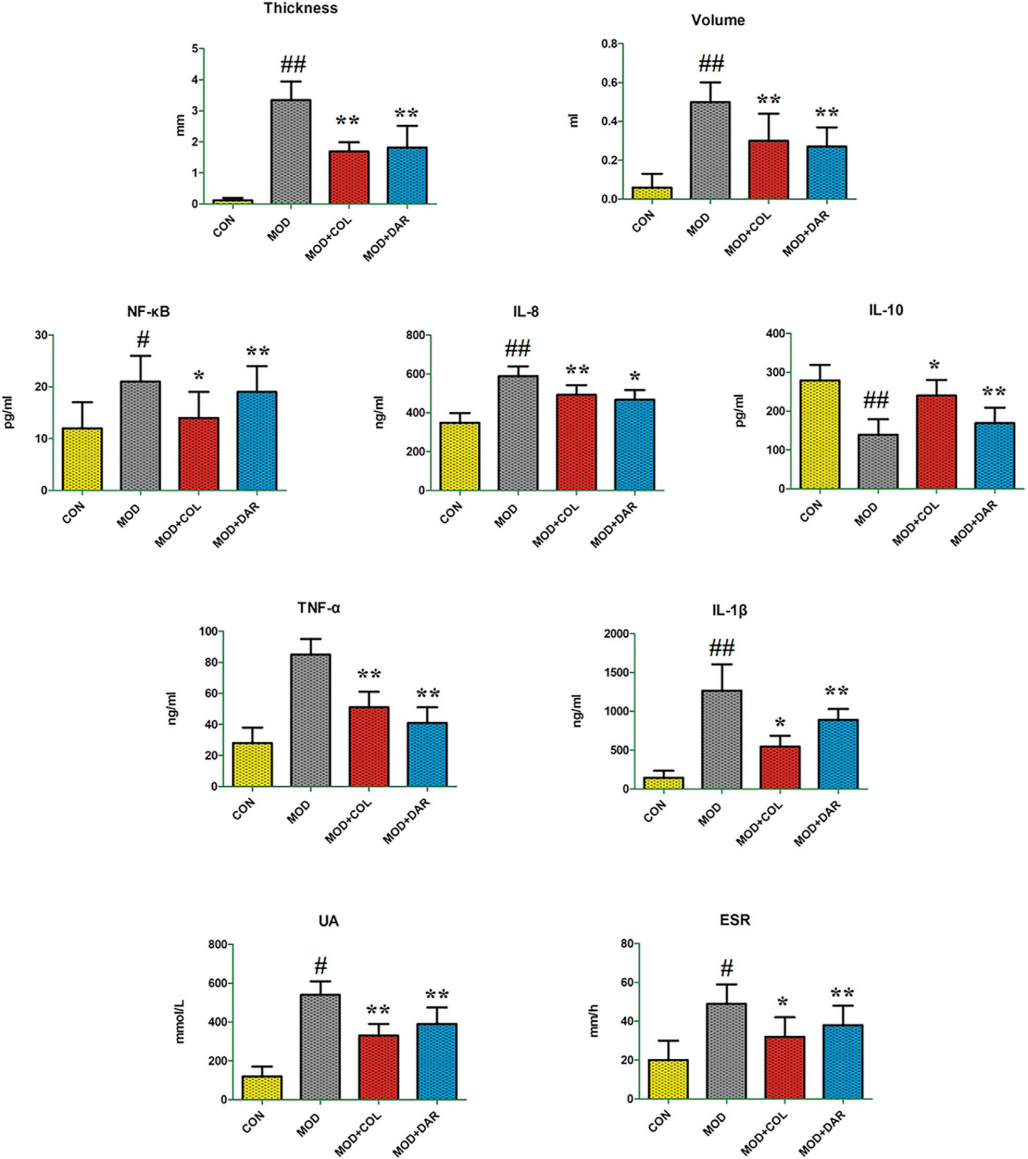
The comparison of physical and chemical indicators changes between CON, MOD, MOD + COL group and MOD + DAR group. Data with statistical significance are represented as “#” or “*” that *p* value less than 0.05. Data with statistical significance are represented as “##” or “**” that *p* value less than 0.01.

The rats in MOD group showed obvious irritability after modeling, distinct redness, swelling, and heat in the right ankle joint accompanied by certain dysfunction, and restricted movement. The rats in CON group did not show obvious irritability. There was no obvious abnormality in the right ankle joint, and their activities were not affected. Before the experiment, the difference in toe volume and right ankle joint thickness of rats in each group was not statistically significant (*p* > 0.05). After modeling, except for the control group, the joints of rats in each group showed different degrees of swelling. Twenty-four hours after modeling, the toe volume and thickness of right ankle joint in MOD group increased significantly compared with those in CON group (*p* < 0.01). Compared with the MOD group, the toe volume and thickness of right ankle joint in MOD + COL group and MOD + DAR group were reduced to varying degrees, and the difference was statistically significant (*p* < 0.01). Compared with the CON group, the content of IL-8, TNF-α, IL-1β, NF-κB, UA in serum and ESR from the MOD group were increased, and IL-10 content were decreased indicating that the acute gouty arthritis model of animals was successfully built. After therapeutic period of darutoside, it could markedly reduce IL-8, TNF-α, IL-1β, NF-κB, UA in serum and ESR with significantly statistical implications (*p* < 0.01or *p* < 0.05). The content of serum IL-10 was significantly higher than those in the MOD group (*p* < 0.01). Biochemical indicators in MOD + COL group and MOD + DAR group showed similar trends, indicating that darutoside has a certain therapeutic effect on acute gouty arthritis, mainly by preventing the inflammatory response, promoting immune system regulation, improve blood circulation (Figure 1).

### Effect of darutoside on metabolomic profiling changes

Under both positive and negative ion modes, blood sample from CON, MOD, MOD + COL and MOD + DAR group could submit good peak shape, temperate intensity and distinct separation, then the metabolic profiling were obtained. In order to estimate instrument precision and method repeatability, before sample list running, the same QC sample was continuously analyzed six times, and six repetitive QC sample were detected at once, which the relative standard deviation (RSD) of ten selected ion peak area and retention time are respectively less than 5% satisfying the analysis requirements. Then, the QC sample was injected once every six experimental samples for continuous tracking of repeatability. Due to spectra complication, multivariate data analysis was performed to amplify and highlight the differences among groups. In Supplementary Figure S2, the PCA score plot distribution in the latent variable space were shown to explain the changed tendency of the CON, MOD, MOD + COL and MOD + DAR groups, which did not exceed the limit indicating anomalous sample was not existed in the clustering of data. The clustering trend of the CON and MOD group were exhibited well separation, and the clustering of MOD + COL and MOD + DAR group remained relatively far from MOD group and close to CON group, suggesting that the model animals of acute gouty arthritis was successfully established and darutoside could improve the abnormal metabolic state to the normal state.

### Effect of darutoside on metabolic biomarker

**FIGURE 2:**
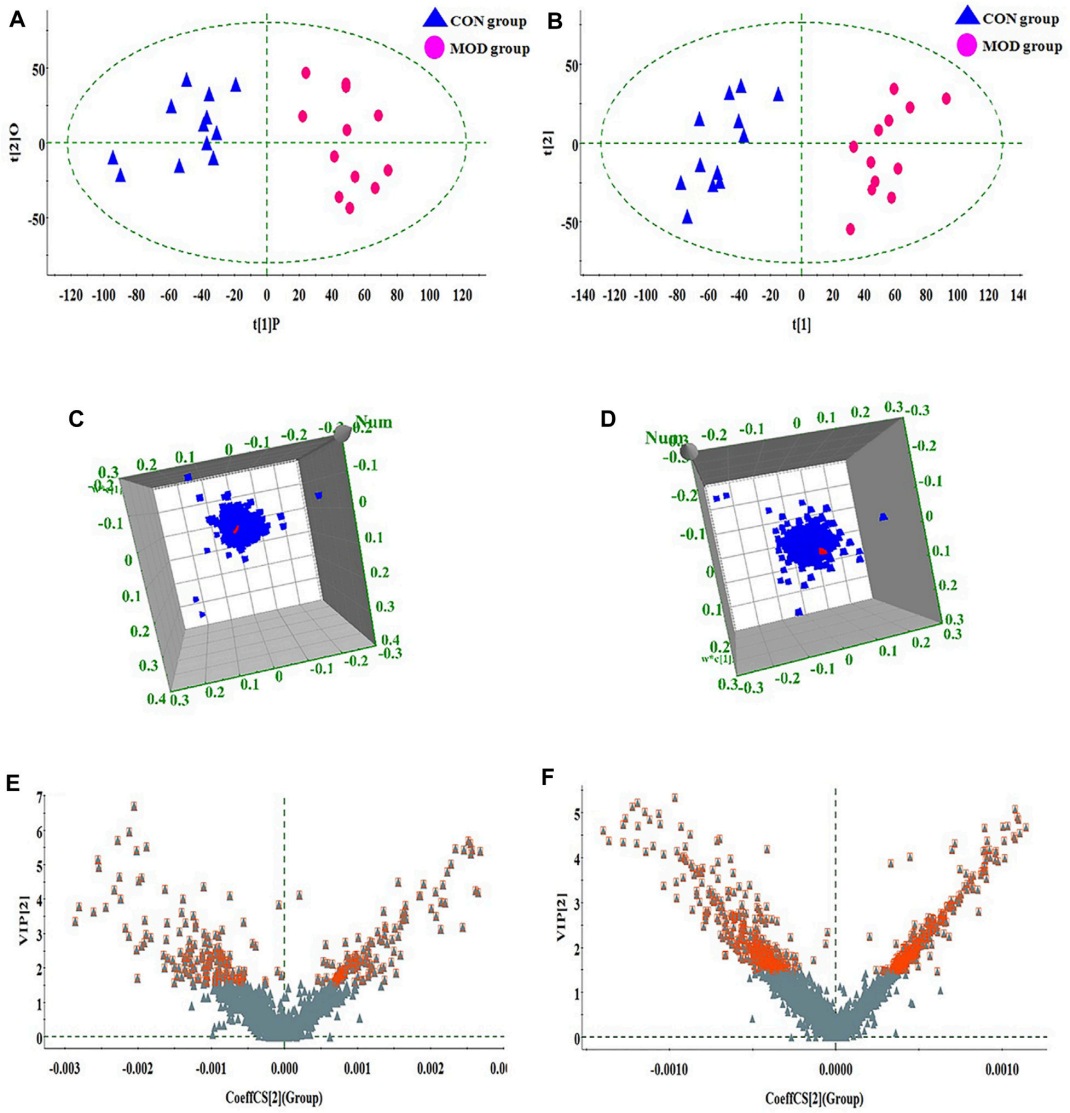
OPLS-DA score plot, 3D loading plot and VIP-plot obtained from CON and MOD group. **(A)**, **(C)** and **(E)** positive ion mode; **(B)**, **(D)** and **(F)** negative ion mode.

**FIGURE 3:**
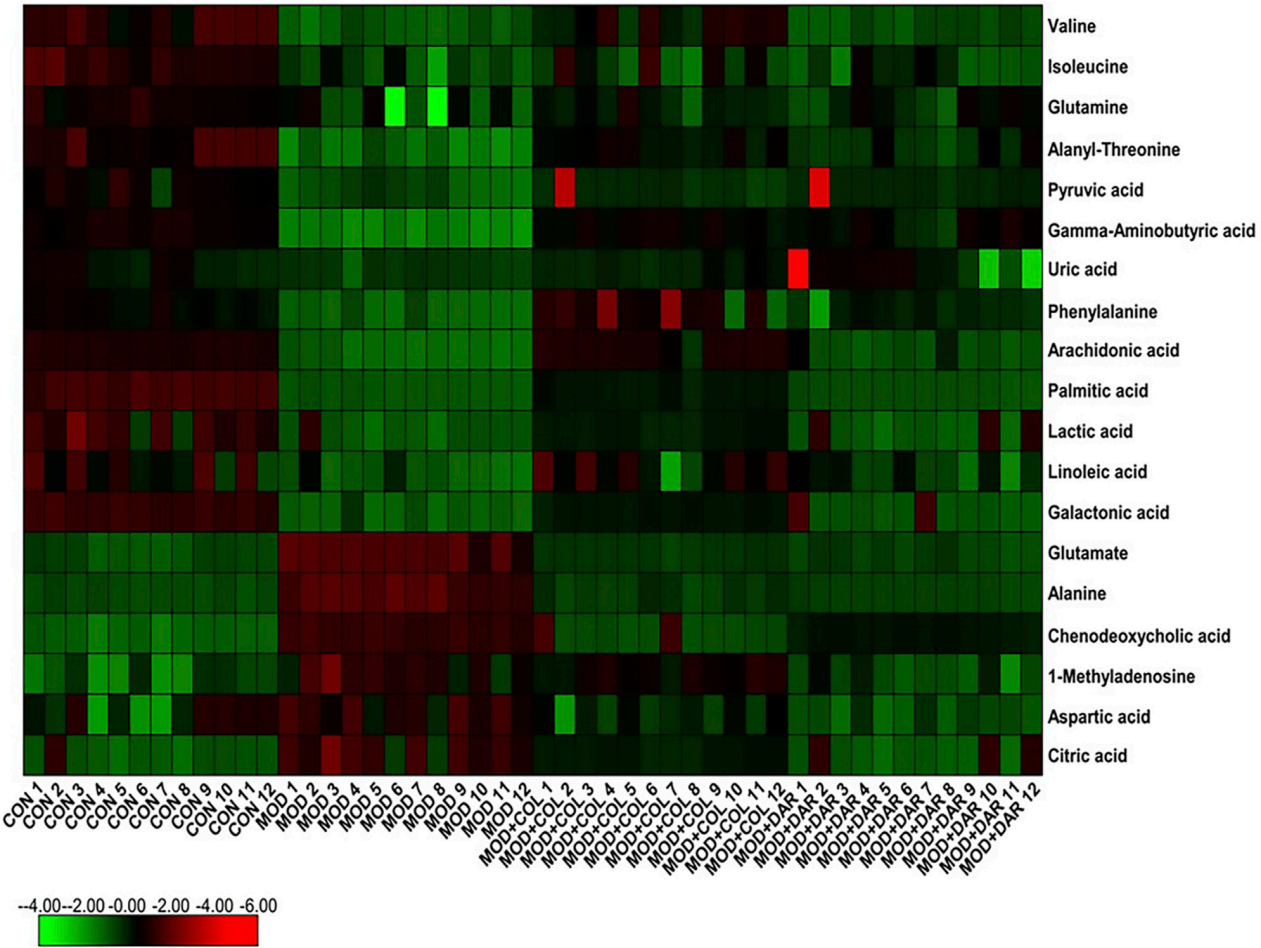
Heatmap illustrating the metabolites changes in serum of CON, MOD, MOD + COL and MOD + DAR group from minimum (dark green) to maximum (dark red) values.

**FIGURE 4:**
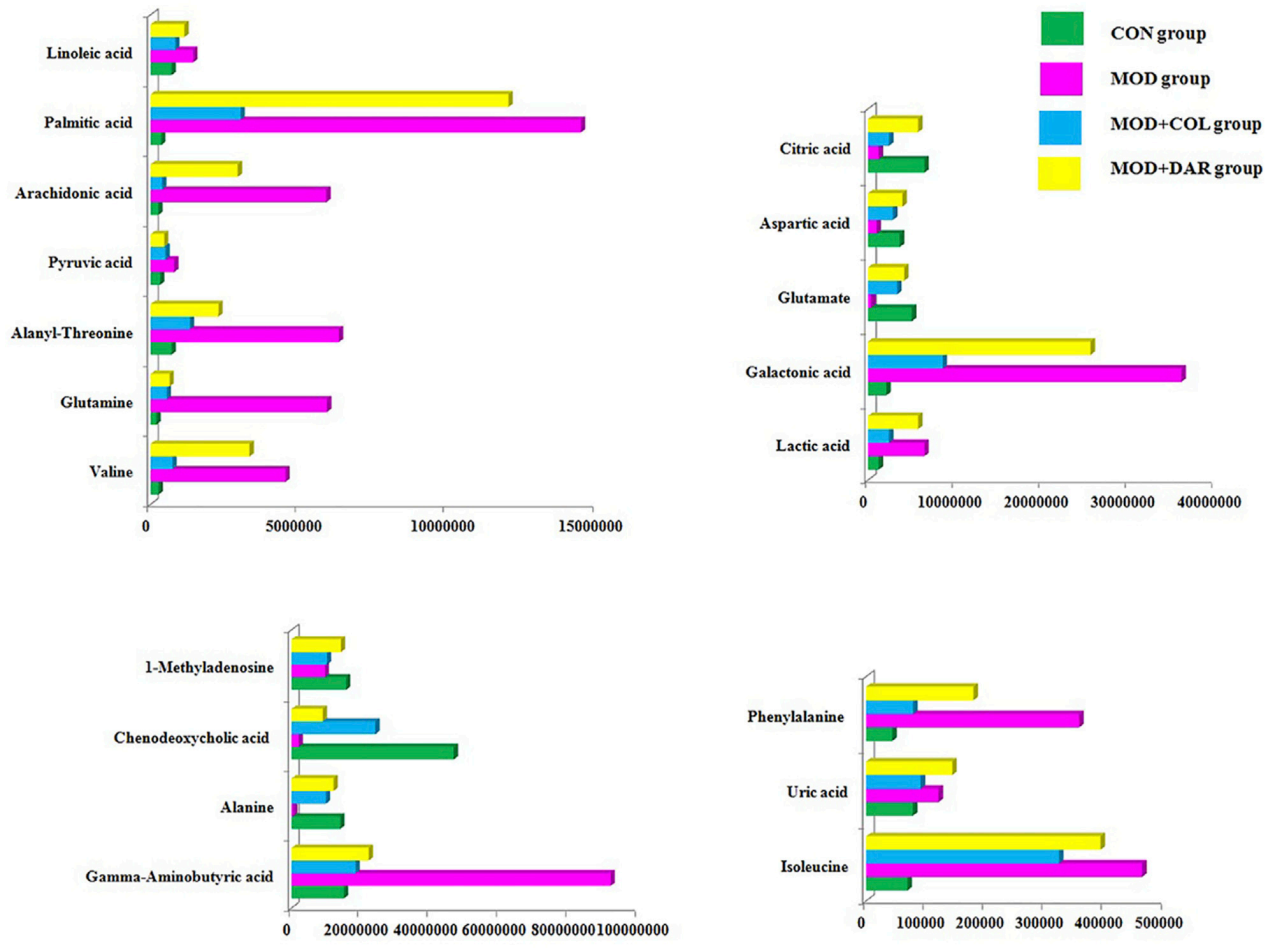
The comparisons of metabolite relative peak area in CON, MOD, MOD + COL and MOD + DAR groups.

In order to seek the differentiated biomarkers, the supervised OPLS-DA models of serum samples between CON and MOD were generated in both ion mode serum samples as displayed in Figures 2A,B [R2Y (cum) = 97.34% and Q2 (cum) = 85.77% in the positive mode, and R2Y (cum) = 98.415 and Q2 (cum) = 90.53% in the negative mode]. The permutation tests (*n* = 200) were used to validate the predictive ability of the gained OPLS-DA models, which the result was showed that all the R2 and Q2 values in OPLS-DA models were lower than in permutation tests indicating the goodness of fit and better predictive capability for the OPLS-DA models. In loading plot of Figures 2C,D, the larger dispersion degree of ions with different mass-to-charge ratio from origin point, the more dramatical contributions to the separation of groups. In VIP volcano plots generated from OPLS-DA model, the variables located on the left top and the right top corner made significant contributions to the separation of CON and MOD group in Figures 2E,F. The differential potential metabolites closely associated with the pathogenesis of acute gouty arthritis in body were selected by synchronously meeting the condition of contribution and variable reliability that VIP score value is more than 1.5 and *p* value is less than 0.05 in Student’s t-test. 29 endogenous metabolites were selected as biomarkers associated with the metabolic disturbances in animals with the acute gouty arthritis in serum sample, including gamma-aminobutyric acid, isocitric acid, valine, linoleic acid, alanyl-threonine, alanine, leucine, 3-hydroxybutyric acid, 3-hydroxyanthranilic acid, citric acid, uric acid, corticosterone, 1-methyladenosine, galactonic acid, glutamine, SM(d18:1/22:0), isoleucine, glutamate, prostaglandin F2a, chenodeoxycholic acid, pyruvic acid, palmitic acid, phenylalanine, arachidonic acid, LysoPC(17:0), aspartic acid, LysoPC(15:0), lactic acid and PE (15:0/20:1), which the main information such as molecular formula, compound name, corresponding m/z, VIP value was enumerated in Supplementary Table S1. After darutoside administration, 19 of above-mentioned metabolites level were regulated closer to normal state, which a heatmap that present the distribution patterns of the above-mentioned metabolites among the CON, MOD, MOD + COL and MOD + DAR groups was shown in Figure 3. The result of horizontal cluster analysis in heatmap is intuitively visualized the pharmacological effect of darutoside on them. The comparisons of metabolite relative peak area in CON, MOD, MOD + COL and MOD + DAR groups are shown in Figure 4. The changed trend of metabolites in MOD + DAR groups was almost the same as MOD + COL group close to the CON group level. Compared to MOD group, the content of glutamate, alanine, chenodeoxycholic acid, 1-methyladenosine, aspartic acid, citric acid were increased, meanwhile, the content of valine, isoleucine, glutamine, alanyl-threonine, pyruvic acid, gamma-aminobutyric acid, uric acid, phenylalanine, arachidonic acid, palmitic acid, lactic acid, linoleic acid and galactonic acid were decreased in the MOD + DAR groups. Among them, the serum level of valine, glutamine, pyruvic acid, uric acid, arachidonic acid, palmitic acid, linoleic acid, galactonic acid, glutamate, chenodeoxycholic acid, aspartic acid and citric acid possess significantly statistical implications (*p* < 0.01) after treatment.

### Effect of darutoside on metabolic pathways

**FIGURE 5:**
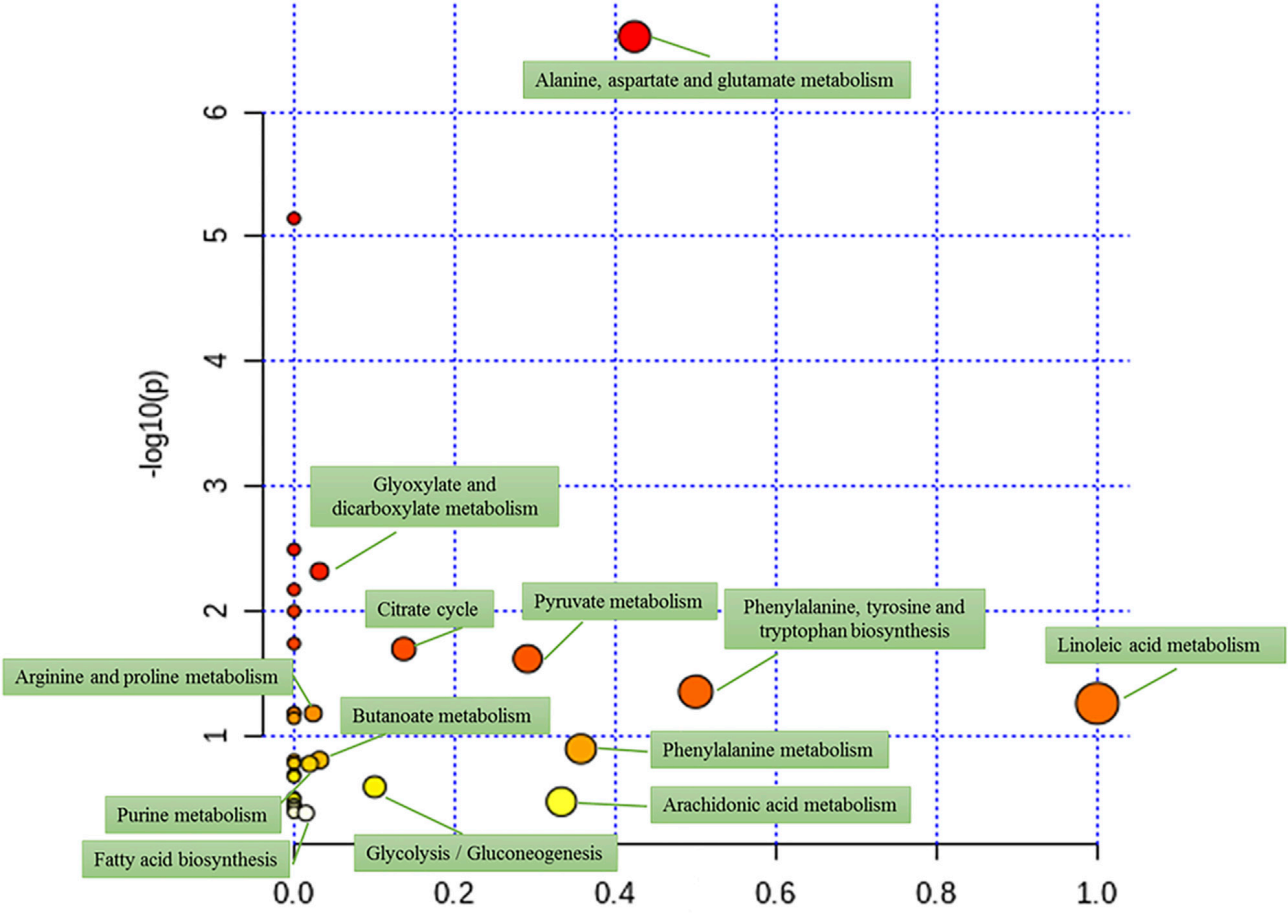
The pathways of disturbed 19 metabolites in response to darutoside treatment.

MetaboAnalyst system was employed to further insight into the metabolic pathways associated with identified biomarkers. Thirteen pathways were obtained in an intuitive manner, including linoleic acid metabolism, phenylalanine, tyrosine and tryptophan biosynthesis, alanine, aspartate and glutamate metabolism, phenylalanine metabolism, arachidonic acid metabolism, pyruvate metabolism, citrate cycle, glycolysis/gluconeogenesis, glyoxylate and dicarboxylate metabolism, butanoate metabolism, arginine and proline metabolism, purine metabolism and fatty acid biosynthesis with the influence value greater than or equal to zero were deemed as the potential vital metabolic pathway of darutoside shown in Figure 5. KEGG global metabolic network of Supplementary Figure S3 provides the important prospecting mapping of metabolites and enzymes/KOs by common metabolomics and metagenimics researches, which amino acid metabolism, glyoxylate and dicarboxylate metabolism, biosynthesis of unsaturated fatty acids, citrate cycle, pantothenate and CoA biosynthesis, pyruvate metabolism were related with anti-inflammation activity of darutoside. Due to similar chemical structures and similar molecular activities, the interaction network of metabolite-metabolite that highlight potential functional relationships between a wide set of annotated metabolites was obtained, and the degree of pyruvic acid, citric acid, alanine, glutamine and aspartic acid was more than 100 degree each other.

### Network construction of “potential metabolite–target–component”

**FIGURE 6:**
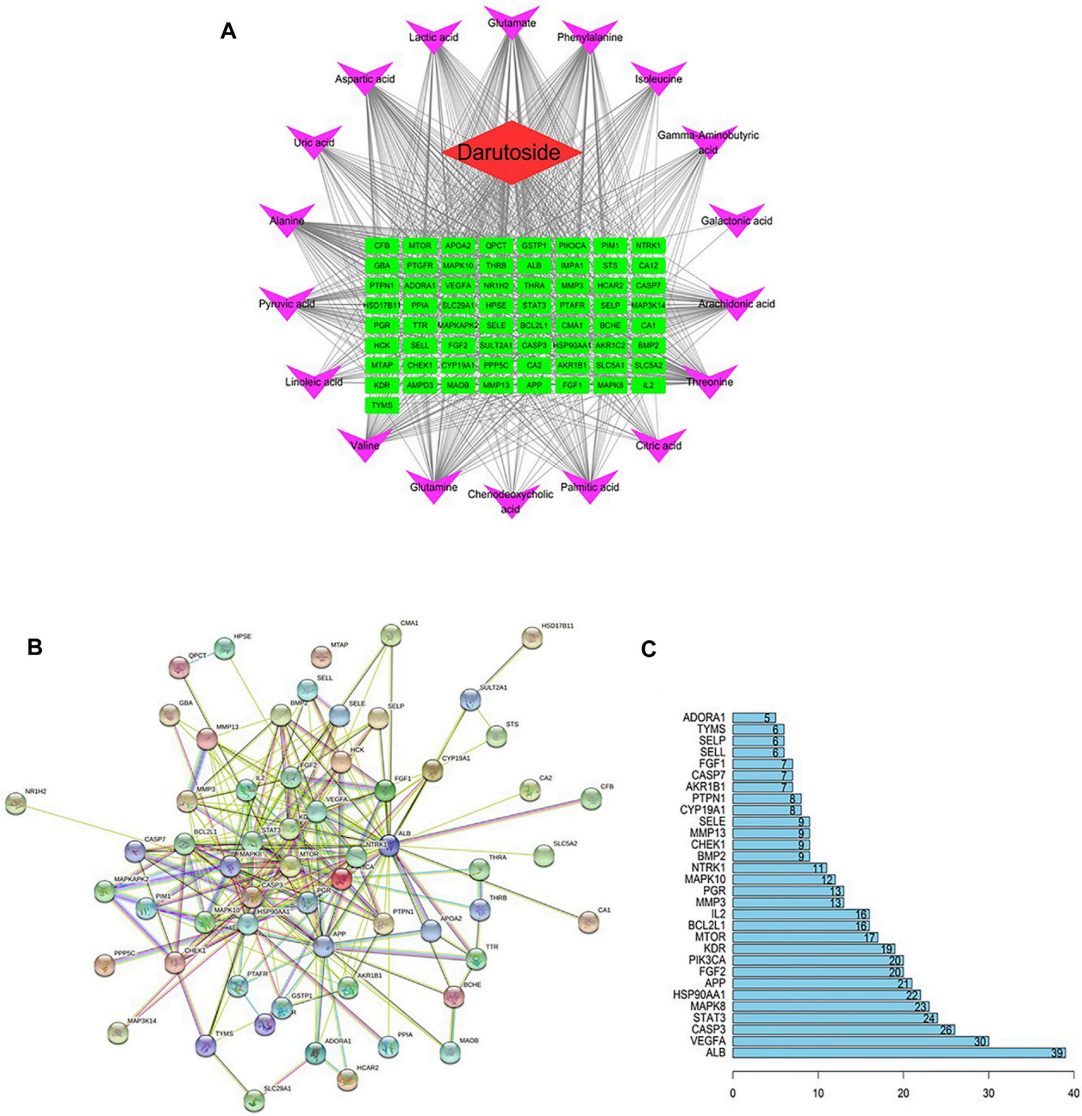
The “ingredients-targets-metabolites” network expressing the interaction among the potential metabolites, protein targets and darutoside regulation **(A)**. Protein-protein interaction network of targets regulated by darutoside **(B)**. The degress value of vital protein targets obtained from protein-protein interaction network**(C)**.

From online database searching, 65 chemical ingredient targets and 55,268 metabolites targets were collected. Among them, 1-Methyladenosine were stoke out due to their targets have nothing to do with darutoside target. The potential metabolite–target–component network as displayed in Figure 6A was indicated that darutoside interacts with 18 serum metabolites and 65 protein targets. Protein-protein interaction network of Figure 6B was consists of 60 nodes and 516 edges, which the larger the node with the darker color and the denser connection is in more important status. ALB, VEGFA, CASP3, STAT3, MAPK8, HSP90AA1, APP, FGF2 and PIK3CA in Figure 6C have more higher correlation degree deemed as potential vital targets for the treatment of acute gouty arthritis, which the relevant node degree value are greater than or equal to 40, respectively.

## Discussion

IL-8 mainly mediates cytotoxicity and local inflammation-related immune response to assist antibody production, and participates in the occurrence of cellular immunity and delayed-type hypersensitivity inflammation. MSU can directly stimulate monocytes in synovial fluid to produce TNF-α. TNF-α is considered to be the first cytokine in the inflammatory cytokine network chain, which can induce other cytokines. Increased expression of TNF-α may enhance the activity of polymorphonuclear leukocytes and expression of IL-1β. Meanwhile, while IL-1β can also increase the activity of TNF-α, and then promotes inflammatory response (Ng et al., 2018). NF-κB usually binds to inhibitor IκB in cells and exists in an inactive state. After being stimulated by TNF-α, it enters the nucleus to initiate transcriptional regulation of cell proliferation and differentiation, and then IKKβ stimulates IkBα phosphorylation and finally activation NF-κB. TNF-α can induce the activation of NF-κB signal transduction pathway in synovial cells, which is closely related to the role of TNF-α in arthritis synovial inflammation and bone destruction (Han et al., 2014). IL-10 as a multi-cell source and functional cytokine regulates cell growth and differentiation, and participates in inflammatory and immune responses. IL-10 inhibits the function of mononuclear macrophages to promote natural and specific immunity, and enhances cell suppression, immune tolerance induction and scavenger functions (Dobrzanski et al., 2012). Blood uric acid exists cell or food in the body, especially foods that is rich in purines. If the body produces too much to be excreted or the uric acid excretion mechanism is degraded, excessive level of uric acid will give rise to gout (Baumgartner et al., 2018). ESR factor is a sign of the sedimentation rate of erythrocytes, which increases significantly in many pathological conditions such as bacterial acute inflammation, hyperglobulinemia, malignant tumors, tissue necrosis as well as anemia (Carpenter et al., 2011).

Another study, the efficacy of Xixiancao (Herba Siegesbeckiae Orientalis) on interactions between nuclear factor kappa-B and inflammatory cytokines in inflammatory reactions of rat synovial cells induced by sodium urate (Wang et al., 2020), was similar to the target and disease species in this study. Among the biochemical indicators of the two studies, NF-κB and IL-1β, IL-8, and TNF-α were selected. The changes in the biochemical indicators of the two studies before and after treatment were consistent, indicating that Herba Siegesbeckiae Orientalis or the main chemical components in it have the same therapeutic effect on gouty arthritis. At the therapeutic level, there are slight differences between the two studies, which may be related to factors such as differences in vivo and *in vitro* models, experimental conditions, and the content of medicinal chemical components.

As a local inflammatory response, acute GA causes severe joint pain and swelling, resulting in changes in endogenous serum metabolite levels. In this study, 29 metabolites related to acute gouty arthritis were identified in MOD group, 19 of which is regulated by darutoside, including glutamate, alanine, chenodeoxycholic acid, 1-methyladenosine, aspartic acid, citric acid, valine, isoleucine, glutamine, alanyl-Threonine, pyruvic acid, gamma-aminobutyric acid, uric acid, phenylalanine, arachidonic acid, palmitic acid, lactic acid, linoleic acid and galactonic acid. The involved metabolic pathways can be divided into five categories: amino acid metabolism pathways (biosynthesis of phenylalanine, tyrosine and tryptophan; metabolism of alanine, aspartic acid and glutamate; metabolism of phenylalanine); Arginine and proline metabolism), sugar metabolism (glycolysis/gluconeogenesis; glyoxylic acid and dicarboxylic acid metabolism), fatty acid metabolism (linoleic acid metabolism, arachidonic acid metabolism, fatty acid biosynthesis), energy metabolism (pyruvate metabolism, citrate cycle), purine metabolism, butyrate metabolism.

There are many amino acids related to immune response in the body. Three common amino acids that constitute proteins in organisms such as leucine, isoleucine and valine are called branched chain amino acids. In addition to promoting metabolism, they also participate in the growth and development of immune organs, immune cells differentiation and division, and immune response regulation to a certain extent. Exogenous uric acid, derived from free purines, purine nucleosides, purine nucleotides, and nucleic acids from food, including various DNA and RNA. Less than 5% of free purines are absorbed into the blood, and only hypoxanthine and xanthine are directly converted to uric acid. Endogenous uric acid comes from the product of purine metabolism, inosincacid can be converted into adenylate and guanylate, or into hypoxanthine, which is then oxidized to xanthine and uric acid. The main amino acids involved in the synthesis of inosincacid are glutamine, aspartic acid, glycine, tryptophan, histidine and serine. If you eat too much food containing the above substances, the body’s uric acid is likely to rise, thereby increasing the risk of gout. Compared with the CON group, the levels of valine and isoleucine in MOD group were increased in this study, indicating that the production of branched chain amino acids can be promoted in the acute gout state (White and Newgard, 2019). Valine and isoleucine facilitate the proliferation of monocytes and humoral immune response. Although glutamate is not an essential amino acid in organisms, it occupies an important position in protein metabolism and belongs to the basic amino acid of nitrogen metabolism (Zhang et al., 2017). Glutamine is not only a source of energy for a variety of immune cells, but also one of the most important immune nutrients, which changed into glutamine under the action of glutamine synthetase. Some researches were reported that glutamine stimulates the proliferation of lymphocytes and macrophages, thereby increasing the content of tumor necrosis factor and white blood cells to enhance the body’s immunity. In this study, the level of glutamate is down-regulated, but the level of glutamine is up-regulated, which is probably caused by the immune system stimulation of the model rats. In order to meet the needs of the body’s immune response, glutamate is converted to glutamine (Chan et al., 2020). The increasing content of threonine that represent immune barrier, immune protein synthesis, protection of lymphocytes, and resistance to free radical damage is closely related to the damage of the immune system (Wang et al., 2010). Sustained high levels of uric acid, energy-compensatory by-products such as uric acid pyruvate and lactate.

Not only participates in the energy metabolism pathway, but pyruvate produces alanine under the catalysis of pyruvate aminotransferase. Studies have shown that the level of alanine aminotransferase in the body can be used to assess tissue damage and adverse events in clinical scientific research trials. In the results of this experiment, the level of pyruvate was up-regulated and alanine was down-regulated, which the activity of alanine transferase in MOD group was reduced, resulting in the synthesis inhibition of alanine (Bernardo et al., 2013; Qiu et al., 2017). In addition, the content of γ-aminobutyric acid as a metabolite related to immunity has also changed, which can also indicate a metabolic disorder of the immune system (McKay et al., 2019). The original metabolism in model rats with acute gouty arthritis is slow, leading to the diseased animals present gasping, coughing, loss of appetite, depression and weight loss, meanwhile, the organism showed an increase in compensatory productivity. Various different metabolites obtained in this result are closely related to energy metabolism. For example, the levels of sugar-generating amino acids such as threonine, valine, isoleucine, and glutamine are up-regulated, and the levels of glutamic acid and aspartic acid are down-regulated, which not only indicates that the amino acid metabolism pathway is disordered, but also the energy metabolism pathway has also been disturbed. Citrate cycle (TCA cycle) is a common core metabolic pathway for the mutual conversion of sugar, fat, and protein, as well as thorough oxidation and decomposition (Hui et al., 2017). Compared with CON group, energy metabolism-related metabolites such as lactic acid, pyruvate, citric acid, and isocitrate level in MOD group has changed. Galactonic acid is the product of the mutual conversion of pentose sugar and glucuronic acid, which forms a spindle group through the oxidation of the hydroxyl group in galactose (Lam and Ng, 2009; Zhang et al., 2016). Galactose is a monosaccharide in dairy products, plant mucins and bacterial polysaccharides. It is an important part of certain glycoproteins and also the main component of lactose in mammalian milk, which can be catalyzed by lactase enzymes in the gut. In the absence of food substrates, uridine diphosphate glucose produces galactose for anabolic metabolism. More importantly, galactoic acid may be associated with D-glucuronic acid and L-galactoic acid, and subsequently is related with pyrimidine metabolism by glucose uridine diphosphate (Zheng et al., 2018). Disorders of carbohydrate metabolism can increase the synthesis of purine substrates and accelerate the production of uric acid, which promote the excretion of phosphorus. Superabundant excretion of phosphorus bring out the burden of glomerular filtration and tubular secretion, and competitively inhibits the excretion of uric acid (Marickar, 2009). In the MOD group, the content of fatty acids metabolites such as palmitic acid, linoleic acid, and arachidonic acid were increased. Fatty acids can generate acetyl coenzymes under sufficient oxygen conditions, and then complete oxidation and decomposition through the TCA cycle to produce CO_2_ and O_2_, which is one of the main sources of cellular energy. In muscles tissue, fatty acids mainly provide energy to tissue cells through the β-oxidation process. Many intermediate products are produced during β-oxidation of fatty acids, such as 3-hydroxybutyric acid and 3-hydroxyanthranilic acid in our research. The abnormal elevated levels of them may be caused by strong β oxidation in the MOD group indicating that the attack of acute gouty arthritis promote fat metabolism, which is in line with the previously proposed compensatory increase in energy production (Liu et al., 2019; Edefonti et al., 2020). Uric acid as a product of purine metabolism is an important part of DNA and RNA. Purine is decomposed into uric acid through a series of catalytic reactions. Pyrimidine metabolism is the first core channel of the entire metabolic network. Guanine can be broken down into uridine monophosphate and uridine diphosphate. Uridine monophosphate is further decomposed into uridine and uracil, then uracil is a unique base of RNA. During DNA transcription, thymine in DNA is substituted and paired with adenine. When the body is in a normal physiological state, purine metabolism is in dynamic balance, and uric acid will not be excessively deposited to induce the formation of gout. In this study, the contents of uric acid, adenosine and glutamine in the purine metabolism pathway of the MOD group were notably changed showing a disorder of purine metabolism in acute gout (Liu et al., 2012; Zhang et al., 2015). Phenylalanine is one of the essential amino acids. When the activity of phenylalanine hydroxylase is reduced or lost, a large amount of phenylpyruvate is produced, which may cause phenylketonuria affecting brain development to cause mental retardation, brain malformations, convulsions and other neurological symptoms. When phenylalanine is lacking in the body, it can lead to insufficient synthesis of tyrosine, then cause hypothyroidism and affect growth. The phenylalanine metabolism level after treatment was significantly reduced, which darutoside showed that could reduce the blood uric acid level by regulating the phenylalanine metabolism (Jiang et al., 2017). Chenodeoxycholic acid is closely related to cholesterol metabolism. Studies have shown that chenodeoxycholic acid can inhibit fibroblasts and intercellular adhesion factors in adjuvant arthritis model rats, which may be related to the transcription level promotion of glucocorticoid receptor mRNA. In addition, chenodeoxycholic acid can improve liver fat and fibrosis, reduce insulin resistance, and has certain effects on diabetic patients, but it is still in clinical trials. Therefore, chenodeoxycholic acid is closely related to the occurrence of hyperuricemia and gout (Wen et al., 2011; Fiorucci and Distrutti, 2019).

Patients with acute gouty arthritis often have gout attacks, and then take drugs, the liver and kidney function damage occurs, the drugs are stopped, and the vicious cycle of gout recurrence and aggravation is performed. The clinical standard research of drug and the evaluation of toxic and side effects are indispensable. Natural medicine protecting against acute gouty arthritis is characterized by “individualization” and “flexibility”, but it has some disadvantages such as inconvenience to take, unclear adverse reactions, lacking clinical recurrence rate and long-term efficacy observation. The research on the mechanism of natural product to prevent and treat gout is the focus of metabolomics in the future, which is more systematic, scientific and prospective. With the development of modern technologies, metabolomics has been widely applied in clinical disease diagnosis, molecular physiopathology, gene functional omics and other fields. Blocking the target cytokines on the signal pathway is the basic way to treat arthritis, and it is also the treatment direction of acute and chronic rheumatic immune system.

## Conclusion

In this study, a combination strategy of metabolomics and network pharmacology were adopted to quest the pharmacological action and effective mechanism of darutoside against rats with acute gouty arthritis. Darutoside has a significant ability to improve the infiltration and release of inflammatory cells, promote blood circulation and improve immunity. It is mediated by regulating 19 differentiated biomarkers related with the pathogenesis of acute gouty arthritis between CON and MOD group, such as glutamate, alanine, chenodeoxycholic acid, 1-methyladenosine, aspartic acid, citric acid level to achieve, which was mainly involved in ameliorating amino acid metabolism pathways, sugar metabolism, fatty acid metabolism, energy metabolis, purine metabolism, butyrate metabolism. In addition, darutoside activity have effect on the expression changes of protein targets such as ALB, VEGFA, CASP3, STAT3, MAPK8, HSP90AA1, APP, FGF2 and PIK3CA from network pharmacology analysis. Darutoside can be used as a novel resource of natural product to treat acute gouty arthritis from multiple links and targets.

## Data Availability

The original contributions presented in the study are included in the article/Supplementary Material, further inquiries can be directed to the corresponding author.
