# Supplementary material for: Revealing the pharmacological effect and mechanism of darutoside on gouty arthritis by liquid chromatography/mass spectrometry and metabolomics
Source: Front Mol Biosci. 2022 Aug 24;9:942303. doi: 10.3389/fmolb.2022.942303 (PMC9448993; doi:10.3389/fmolb.2022.942303)
Supplement: Supplementary file 1 [file DataSheet1.docx]

**Table S1.**  Differential expressed metabolites of rats with acute gouty arthritis before and after darutoside treatment using high-throughput LC-MS analysis.

| **No.** | **Scan Mode** | **t_R_** | **Name** | **Proposed compound** | **m/z determined** | **HMDB** | **VIP value** | **Trend in MOD** | **DAR treatment** |
| --- | --- | --- | --- | --- | --- | --- | --- | --- | --- |
| 1 | M+H | 0.52 | Gamma-Aminobutyric acid | C4H9NO2 | 104.0709 | HMDB00112 | 2.05 | ↑ | ● |
| 2 | M-H | 0.72 | isocitric acid | C6H8O7 | 191.0196 | HMDB00193 | 3.03 | ↓ |  |
| 3 | M+H | 1.51 | Valine | C5H11NO2 | 118.0862 | HMDB00883 | 2.45 | ↑ | ● |
| 4 | M+H | 1.68 | Linoleic acid | C18H32O2 | 281.2481 | HMDB0000673 | 3.34 | ↑ | ● |
| 5 | M+H | 1.79 | Alanyl-Threonine | C7H14N2O4 | 191.1016 | HMDB28697 | 2.75 | ↑ | ● |
| 6 | M+H | 2.14 | Alanine | C3H7NO2 | 90.0553 | HMDB00161 | 3.23 | ↓ | ● |
| 7 | M+H | 2.27 | Leucine | C6H13NO2 | 132.1025 | HMDB0000687 | 5.55 | ↓ |  |
| 8 | M-H | 2.56 | 3-Hydroxybutyric acid | C4H8O3 | 103.0390 | HMDB00357 | 7.54 | ↑ |  |
| 9 | M+H | 3.17 | 3-Hydroxyanthranilic acid | C7H7NO3 | 171.0769 | HMDB0001476 | 2.10 | ↑ |  |
| 10 | M+H | 3.33 | Citric acid | C6H8O7 | 215.0170 | HMDB0000094 | 2.89 | ↓ | ● |
| 11 | M+H | 3.84 | Uric acid | C5H4N4O3 | 169.0360 | HMDB00289 | 2.69 | ↑ | ● |
| 12 | M-H | 4.02 | Corticosterone | C21H30O4 | 345.2052 | HMDB01547 | 3.05 | ↓ |  |
| 13 | M-H | 4.54 | 1-Methyladenosine | C11H15N5O4 | 280.1064 | HMDB03331 | 2.68 | ↓ | ● |
| 14 | M-H | 4.78 | Galactonic acid | C6H12O7 | 195.0507 | HMDB0000565 | 3.34 | ↑ | ● |
| 15 | M-H | 4.82 | Glutamine | C5H10N2O3 | 145.0613 | HMDB00641 | 9.25 | ↑ | ● |
| 16 | M-H | 5.07 | SM(d18:1/22:0) | C45H91N2O6P | 785.6510 | HMDB12103 | 1.69 | ↓ |  |
| 17 | M+H | 5.38 | Isoleucine | C6H13NO2 | 132.1005 | HMDB00172 | 2.75 | ↑ | ● |
| 18 | M-H | 5.55 | Glutamate | C5H9NO4 | 146.0460 | HMDB0000641 | 3.00 | ↓ | ● |
| 19 | M-H | 6.12 | Prostaglandin F2a | C20H34O5 | 399.2419 | HMDB0001139 | 4.25 | ↑ |  |
| 20 | M+H | 6.45 | Chenodeoxycholic acid | C24H40O4 | 393.5720 | HMDB00518 | 1.84 | ↓ | ● |
| 21 | M-H | 6.69 | Pyruvic acid | C3H4O3 | 87.0079 | HMDB00243 | 4.38 | ↑ | ● |
| 22 | M-H | 6.83 | Palmitic acid | C16H32O2 | 255.2300 | HMDB0000220 | 1.79 | ↑ | ● |
| 23 | M+H | 7.36 | Phenylalanine | C9H11NO2 | 166.0848 | HMDB00159 | 2.64 | ↑ | ● |
| 24 | M+H | 7.42 | Arachidonic acid | C20H32O2 | 327.2304 | HMDB01043 | 1.52 | ↑ | ● |
| 25 | M-H | 7.86 | LysoPC(17:0) | C25H52NO7P | 508.3428 | HMDB12108 | 2.69 | ↓ |  |
| 26 | M+H | 8.11 | Aspartic acid | C4H7NO4 | 134.1027 | HMDB0000191 | 1.89 | ↓ | ● |
| 27 | M+H | 8.29 | LysoPC(15:0) | C23H48NO7P | 482.3245 | HMDB10381 | 7.86 | ↓ |  |
| 28 | M-H | 9.33 | Lactic acid | C3H6O3 | 89.0234 | HMDB00190 | 2.33 | ↑ | ● |
| 29 | M+H | 9.56 | PE(15:0/20:1) | C40H78NO8P | 732.5558 | HMDB08900 | 3.44 | ↓ |  |


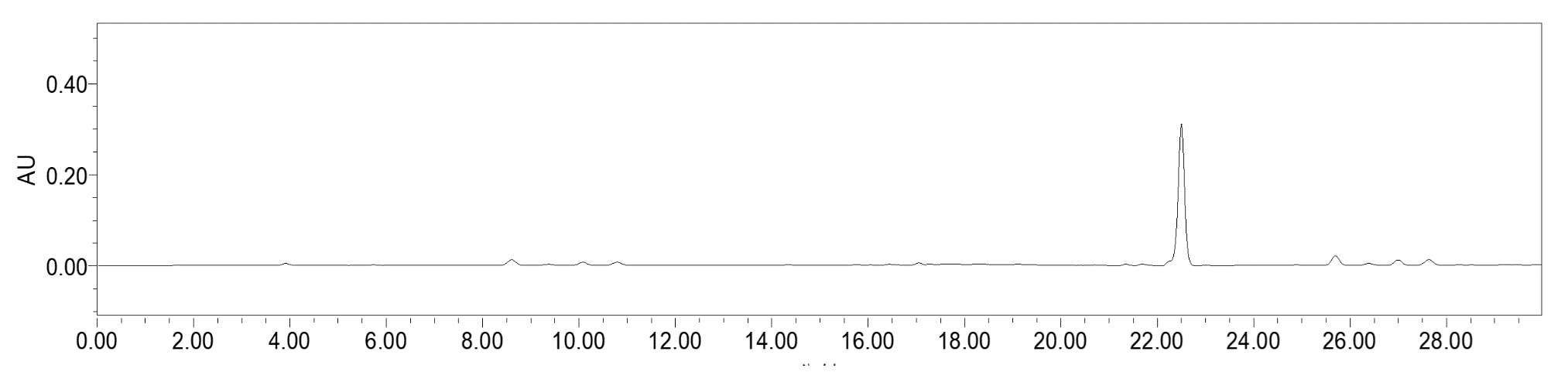


Figure S1. The HPLC Chromatogram of darutoside

The chromatographic column is phenomenex C18 column (250 mm × 4.6mm, 5 μ m) ; mobile phase: acetonitrile methanol-0.05 mol/L potassium dihydrogen phosphate aqueous solution (24 ∶ 6 ∶ 70); volume flow: 1.0 ml/min; column temperature: 30 ° C; detection wavelength: 215 nm; injection volume: 20 μ L。


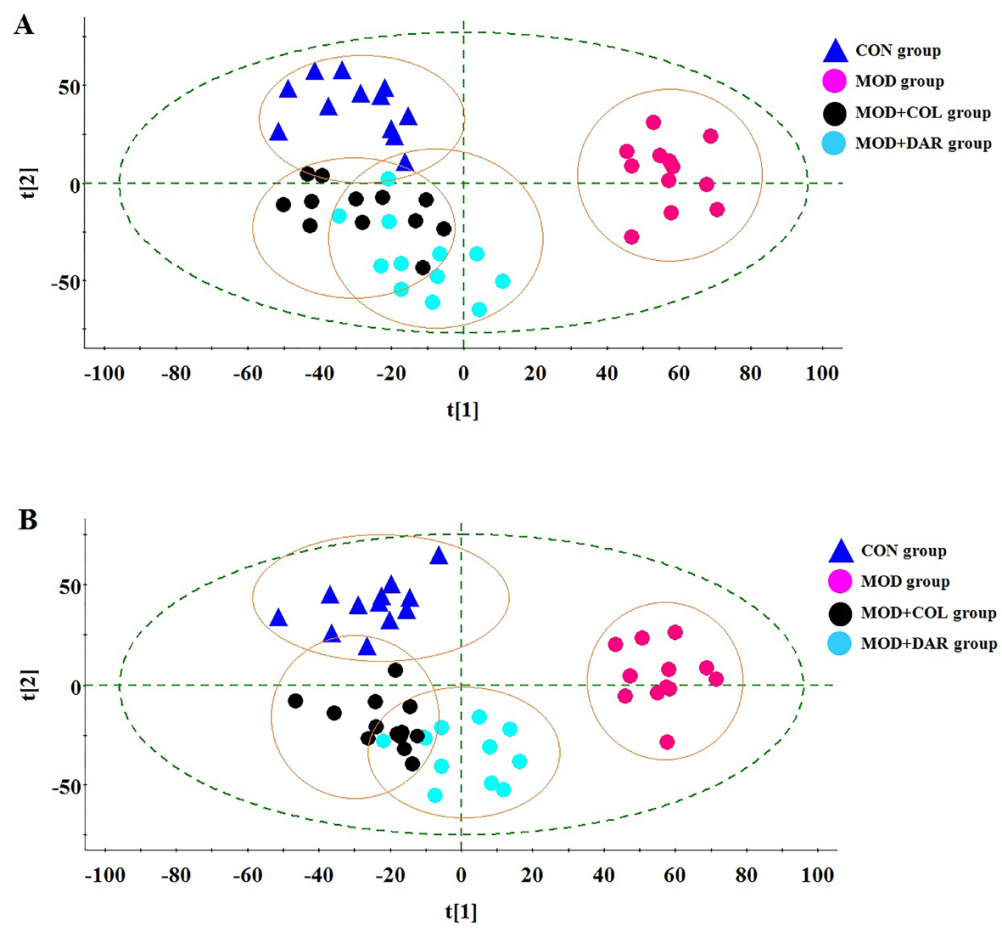


Figure S2


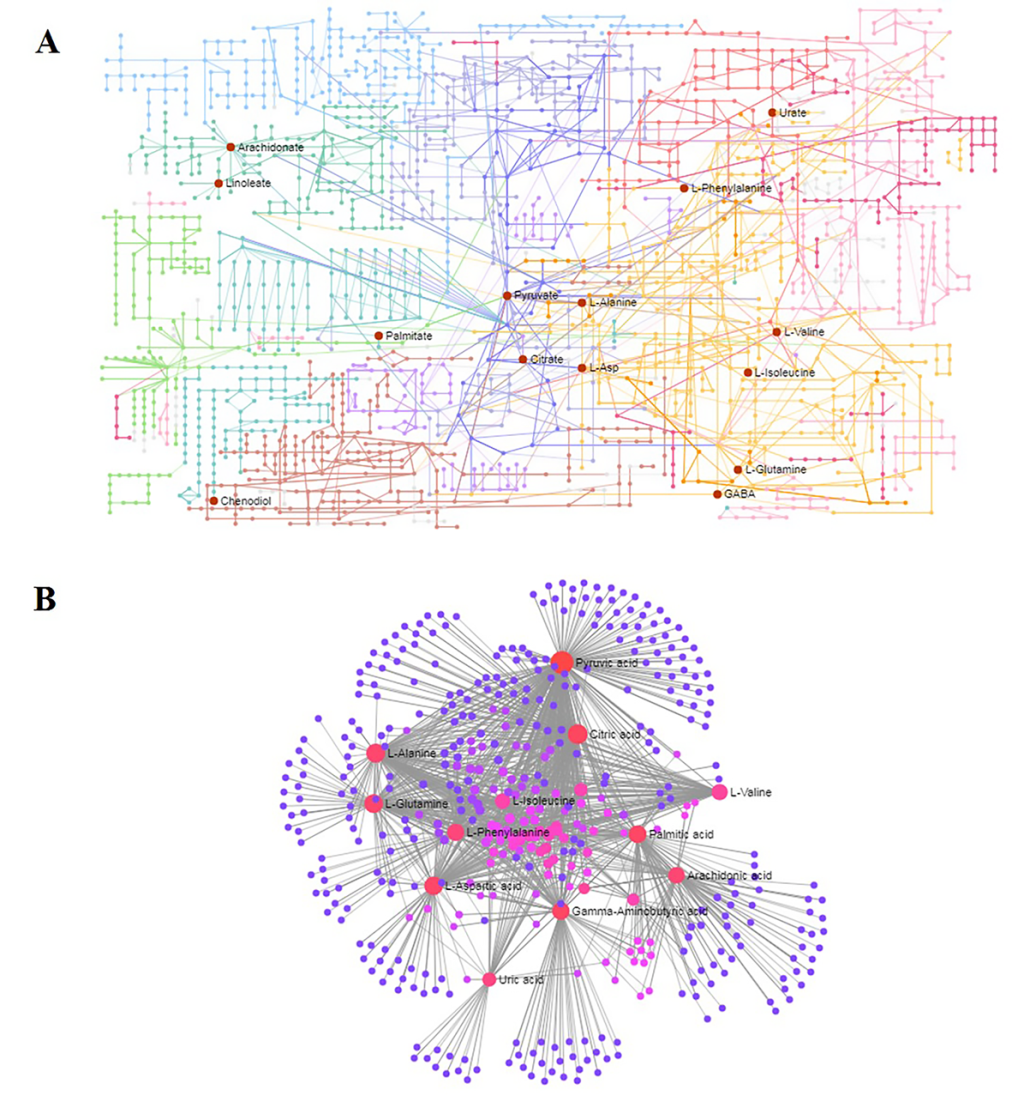


Figure S3
